# Mitochondria in Pathological Cardiac Hypertrophy Research and Therapy

**DOI:** 10.3389/fcvm.2021.822969

**Published:** 2022-01-18

**Authors:** Dan Yang, Han-Qing Liu, Fang-Yuan Liu, Zhen Guo, Peng An, Ming-Yu Wang, Zheng Yang, Di Fan, Qi-Zhu Tang

**Affiliations:** ^1^Department of Cardiology, Renmin Hospital of Wuhan University, Wuhan, China; ^2^Hubei Key Laboratory of Metabolic and Chronic Diseases, Wuhan, China; ^3^Department of Thyroid and Breast, Renmin Hospital of Wuhan University, Wuhan, China

**Keywords:** mitochondria, cardiac hypertrophy, regulatory mechanisms, therapeutic strategies, mitophagy

## Abstract

Cardiac hypertrophy, a stereotypic cardiac response to increased workload, ultimately progresses to severe contractile dysfunction and uncompensated heart failure without appropriate intervention. Sustained cardiac overload inevitably results in high energy consumption, thus breaking the balance between mitochondrial energy supply and cardiac energy demand. In recent years, accumulating evidence has indicated that mitochondrial dysfunction is implicated in pathological cardiac hypertrophy. The significant alterations in mitochondrial energetics and mitochondrial proteome composition, as well as the altered expression of transcripts that have an impact on mitochondrial structure and function, may contribute to the initiation and progression of cardiac hypertrophy. This article presents a summary review of the morphological and functional changes of mitochondria during the hypertrophic response, followed by an overview of the latest research progress on the significant modulatory roles of mitochondria in cardiac hypertrophy. Our article is also to summarize the strategies of mitochondria-targeting as therapeutic targets to treat cardiac hypertrophy.

## Introduction

Cardiomyocytes (CMs) are one of the most important cell types which become terminally differentiated once after birth. Therefore, in adult hearts, CMs have no capability of proliferating in response to prolonged pressure/volume overload; instead, they exhibit morphological enlargement to continuously pump blood to provide oxygen and nutrients to the body, thus leading to the increased heart wall thickness and heart mass, and eventually cardiac hypertrophy (1). Cardiac hypertrophy can be generally divided into physiological and pathological hypertrophy. Physiological hypertrophy, which usually occurs during pregnancy and exercise, is characterize with a coordinated increase in ventricular volume and wall thickness, most importantly, it is reversible once the stimulus was relieved (2). In contrast, pathological hypertrophy, a decompensated stage with complex network of cellular and molecular regulation, usually observed in patients with long-term cardiovascular disorders, is accompanied by cardiac systolic/diastolic dysfunction and the enlargement of CMs is often irreversible (3, 4). Pressure/volume overload, Angiotensin II (Ang II), oxidants, activation of α/β adrenergic receptors, hypoxia, aging, and high-glucose are efficient inducers of pathological cardiac hypertrophy (5). It is well established that pathological cardiac hypertrophy has become an independent risk factor for the development of heart failure (HF), a major and growing public health problem globally with increasing morbidity, high mortality, and heavy economic burdens (6).

Over the past few decades,extensive studies have established that previously unrecognized mechanisms, including epigenetic modifications, immunomodulation, impaired protein quality control, aberrant Ca2+ handling, metabolic reprogramming, and cell-to-cell interactions, are involved in the initiation and progression of cardiac hypertrophy (5). With the continuous deepening of research and updating of knowledge, energy metabolism has shown an increasingly compelling role in cardiac hypertrophy. It is widely accepted that mitochondria serve as the power-house of cells and mitochondrial dysfunction often highly correlates with cell pathology, especially in cells requiring high-energy supply such as CMs. Morphometry analysis has demonstrated that the contents of mitochondria occupy 22.0-37.0% of the CMs' mass in various species, and most importantly, the volume densities of mitochondria are closely related to heart rate and cardiac oxygen consumption rate (7). It is also estimated that 60–90% of the energy adult hearts required is originated from the oxidation of fatty acid in mitochondria (8). Therefore, mitochondria serve as the central organelle responsible for coordinating the energy transduction and maintaining the contractile performance of CMs. The critical roles of mitochondria in cardiac ischemia-reperfusion injury, acute myocardial infarction, atherosclerosis, and cardiac aging have recently been reviewed detailedly (9–12). During hypertrophy, mitochondria exhibit extensive alterations in morphology, biogenesis, dynamics and bioenergetics, and mitochondria also participate in the modulation of hypertrophic response and contribute to the transition to decompensated heart failure (13). Mitochondrial abnormalities and myocardial hypertrophy are intimately linked and deserve attention.

In this review, we will summarize the morphological and functional changes of mitochondrial that occur in the hypertrophic hearts, followed by an overview of the critical modulatory roles of mitochondria in response to hypertrophic stimuli, and finally, we will present and discuss the potential therapeutic strategies to prevent or reverse cardiac hypertrophy.

## Mitochondrial Alterations Through the Transition from Cardiac Hypertrophy to Failure

In hearts of Ang II-induced left ventricular hypertrophy mice, reactive oxygen species (ROS) production, mitochondrial protein carbonyls, mitochondrial DNA damage, signaling related to mitochondrial biogenesis, and autophagy show a dramatic increase (14). Furthermore, in right ventricular cardiac hypertrophy and HF, mitochondrial morphology, fusion/fission balance, Ca^2+^ kinetics, and supercomplex activity are markedly altered, and the dysfunction of mitochondria is mainly attributed to the dysfunction of complex I (15). Increasing lines of evidence demonstrate that during the hypertrophic response, mitochondria display extensive alterations in both morphology and function, which, in turn, directly or indirectly influence the progression of pathological hypertrophy (5, 16). In this section, we will give a summary of the most recent research progress on mitochondrial changes, mainly morphologically and functionally, in response to cardiac hypertrophy.

### Mitochondrial Morphological Alterations

Mitochondria play a central role in both cellular physiology and pathology. The morphology of mitochondria is highly plastic, which to some extent is delicately regulated by mitochondrial biogenesis, fission and fusion (17). Biogenesis is usually provoked by preexisting abnormal mitochondria, and it is a complex process during which the mitochondria undergo growth and division with changes in mitochondrial number, size and mass (18). Mitochondrial fission and fusion are collectively termed “mitochondrial dynamism,” and genetic interruption of either triggers an accumulation of damaged mitochondria and results in severe cardiomyopathy (19).

Mitochondria in healthy hearts are relatively rich and intact, with complete membrane structures and clear cristae structures; while in hypertrophic hearts, the mitochondria become seriously swollen and deformed, with blurred and ruptured membrane and cristae structures (20). Besides, mitochondria in normal CMs appear to be columnar/ovate in shape, but become filamentous/elongated under stress (20). The morphometric parameters indicated that persistent overload could elevate the area, diameter and perimeter of mitochondria and decrease mitochondrial volume density (21). Utilizing confocal and electron microscopy, researchers have also revealed that in rat models of HF, there exists an increase in small and fragmented mitochondria, which is consistent with an imbalance between mitochondria fusion and fission (22). Mitochondrial cristae, the true bioenergetic components of cells, display prominent morphological changes in the process of cardiac hypertrophy. A recent study has demonstrated that abundant fragmented and lysed cristae was observed in palmitate-treated neonatal rat cardiomyocytes (NRCMs), accompanied by decreased mitochondrial network and complex expression, and elevated ROS levels, which ultimately contribute to NRCM enlargement; and adenovirus-mediated mitofilin overexpression promisingly restored cristae shape and cardiac hypertrophy (23). Therapeutic strategies targeting cristae remodeling may represent an effective way to protect against cardiac hypertrophy.

Interestingly, immunofluorescence staining illustrated a preferred perinuclear localization of mitochondria in normal NRCMs, while in NRCMs stimulated with PE, mitochondria were more dispersed and extensively distributed in the cytoplasm of the cell (24). Gene array analysis suggested that kinesin family member 5b (Kif5b), a kinesin motor protein, was differentially expressed under PE stimulatory conditions. Depletion of Kif5b through specific siRNA targeting Kif5b prevented the peripheral mitochondrial localization in NRCMs as well as reverted PE-induced hypertrophy (24).

Taken together, the data support significant changes in mitochondrial morphology under the stimulation of cardiac hypertrophy.

### Mitochondrial Functional Alterations

Luo and colleagues have systematically analyzed the cellular and molecular transcriptomic landscape in non-failing (NF) and heart failure with reduced ejection fraction (HFrEF) human hearts. Ingenuity Pathway Analysis (IPA) revealed extensive transcriptional and signaling changes in HFrEF tissues. Notably, the left ventricles (LV) of the failing hearts displayed prominently altered mRNA transcripts related to mitochondrial permeability and mitochondrial biogenesis, including *BCL2L1, CAV2, COX10, DNAJA3, HSPA5, KRT8, PRDX3, PTCD2*, and etc. (25). These dramatic HFrEF-related transcriptional alterations in the left heart suggested a reciprocal relationship between mitochondrial dysfunction and cardiac pathology.

In the early stage of the development of pathological cardiac hypertrophy, remarkable cardiac insulin-resistance, decreased mitochondrial complex V activity and significantly impaired mitochondrial energy metabolism were observed, which implied that early mitochondrial dysfunction and energy deficit may contribute to the transition from adaptive to maladaptive cardiac hypertrophy (26). Similarly, by combining the transcriptome of individual CMs with cellular morphology, epigenomic characteristics and cardiac function, Nomura et al. successfully reconstructed the trajectory of cardiac hypertrophy and failure, and they demonstrated that in the early stage of cardiac hypertrophy, a number of genes encoding proteins which function in mitochondrial translation/metabolism were significantly activated in CMs (27). Additionally, mitochondria's ability to produce ATP is significantly impaired in response to cardiac hypertrophy. *In vivo* experimental data showed that the activities of NADH dehydrogenase, succinate dehydrogenase (SDH), and mitochondrial membrane potential (MMP) were significantly reduced after hypertrophic insults (21). *In vitro* experiments also suggested that H9c2 cells treated with isoproterenol (ISO) exhibited ROS overload, NO/ROS imbalance, and reduced MMP (28).

The metabolic and transcriptional profiles of hypertrophic murine hearts induced by TAC contribute to a better understanding of the metabolic alterations during maladaptive hypertrophy. By performing microarray analysis, Frey' group have revealed that, in the hearts 6 weeks after TAC surgery, genes involved in mitochondrial beta-oxidation were markedly downregulated, in addition, the expression of certain key enzymes implicated in oxidative phosphorylation and citrate cycle also showed a significant decrease, indicating disturbed mitochondrial function during transition to heart failure (29). And notably, transgenic mice overexpressing catalase in the mitochondria, instead of peroxisomes, were protected from pathological cardiac hypertrophy induced by Ang II stimulation, suggesting that ROS produced in mitochondria plays a central role in mitochondrial energetic failure during decompensated hypertrophy (14).

In summary, it's reasonable to conclude that mitochondrial dysfunction occurs in the early stage of compensatory cardiac hypertrophy, and the functional alteration of mitochondria may represent one essential pathophysiological process that contributes to the transition from compensatory cardiac hypertrophy to decompensated heart failure (Table 1).

**Table 1:** Key features of cardiac hypertrophy and the mitochondrial alterations.

	**Cardiac homeostasis**	**Cardiac hypertrophy**
Stimuli	None	Cardiac volume/pressure overload, Ang II, oxidants, activation of α/β adrenergic receptors, hypoxia, aging, high-glucose, and etc.
Cardiac structure	Normal	Enlarged cardiomyocytes, increased heart wall thickness and heart mass, interstitial fibrosis, and increased cellular apoptosis
Cardiac function	Normal	Impaired
Mitochondrial structure	Rich and intact, with complete membrane structures and clear cristae structures	Swollen and deformed, with blurred and ruptured membrane and cristae structures
Mitochondrial distribution	Perinuclear	Dispersed, mainly distributed in the cytoplasm of the cell
ATP production	Normal	Impaired
ROS generation	Normal	Increased
Fatty acid oxidation	Normal	Decreased
Mitochondrial membrane potential (MMP)	Normal	Decreased
Ca^2+^-induced mitochondrial permeability transition pore	Normal	Increased
NADH dehydrogenase activity	Normal	Decreased
Succinate dehydrogenase (SDH) activity	Normal	Decreased
Mitochondrial dynamics	Normal	Imbalance in mitochondrial fission and fusion
Mitochondrial biogenesis	Normal	Insufficient
Mitophagy	Normal	Transiently activated 3–7 days post-TAC, and downregulated thereafter

## Mechanisms Linking Mitochondria and Pathological Cardiac Hypertrophy

### Mitochondrial Dynamics

As a dynamic organelle, the mitochondria constantly remodel and exchange contents during periodic fission and fusion. The homeostasis of mitochondrial dynamics is accurately regulated by mitochondrial fission and fusion factors (30). Previous studies have established that the ablation of mitofusin (Mfn) 2 interrupted mitochondrial fusion and caused an accumulation of mitochondria with morphological and functional abnormalities, eventually resulting in progressive cardiac hypertrophy (31). Disruption of mitochondrial fission mediated by Dynamin-related protein1 (Drp1), a pro-fission protein that opposes Mfn2 in mitochondrial dynamics, led to elongated mitochondria, elevated mitochondrial contents, mitochondrial dysfunction, and perturbed mitophagy; furthermore, Drp1-deficient mice developed cardiac pathology and displayed worsened cardiac function (32, 33). Notably, interruption of Mfn1/Mfn2-mediated mitochondrial fusion or Drp1-mediated mitochondrial fission in adult murine hearts was related to rapid lethality (32, 34). Utilizing cardiac-specific deletion of Mfn1/Mfn2 and Drp1 to simultaneously eliminate mitochondrial fission and fusion in the hearts of adult mice, researchers revealed that Mfn1/Mfn2/Drp1 cardiac TKO (triple knockout) mice had a longer survival time and developed a unique form of pathological cardiac hypertrophy compared with fusion-defective Mfn1/Mfn2 cKO or fission-defective Drp1 cKO mice, and provided evidence that cardiac pathology was attributed to the imbalance in mitochondrial dynamism (fission and fusion), instead of the absence of either fission or fusion (19). Thus rebalancing mitochondrial dynamics constitutes a therapeutic candidate to ameliorate cardiac cardiomyopathies which were provoked by imbalanced mitochondrial fission/fusion.

### Mitochondrial Calcium Uptake

Calcium ion (Ca^2+^) plays a central role in mitochondrial function in the heart. Mitochondrial Ca^2+^ intake contributes to the generation of ATP and helps to maintain myocardial contractility. Notably, excessive or insufficient mitochondria Ca^2+^ in the CMs leads to mitochondrial dysfunction and cardiac pathology (35, 36). Recent studies have suggested that, by delicately regulating the uptake and release of Ca^2+^ in response to hypertrophic stimuli, mitochondria maintain the Ca^2+^ homeostasis and cardiac function.

Voltage-dependent anion channel 2 (VDAC2), a porin present on the outer mitochondrial membrane, has recently been reported of high importance to control cardiac remodeling (37). Shankar and co-workers successfully performed cardiac-specific *Vdac2* knockout mice and demonstrated that, VDAC2-KO mice exhibited progressive deterioration of cardiac function and eventually developed dilated cardiomyopathy (DCM). Gene and protein expression profiles of the hearts from 16-week-old VDAC2-KO mice suggested that calcium signaling and regulation were markedly altered. In addition, impaired calcium cycling, increased cardiac fibrosis, metabolic alterations were observed in KO mice. Transmission electron microscopy (TEM) was also performed and substantial alterations in the distribution and structure of mitochondria were observed; generally, loss of VDAC2 caused prominently smaller, less dense, and disoriented mitochondria compared to control. Promisingly, reintroduction of VDAC2 through injection with AAV9-αMHC-VDAC2-GFP vector aided in cardiac structural and functional improvement in VDAC2-KO mice. Administration of efsevin, a VDAC2 agonist, greatly enhanced contractile force and accelerated relaxation in the failing hearts, thus VDAC2 may constitute a candidate for HF therapy. Mitochondrial calcium uptake 1 (MICU1), a Ca^2+^ binding protein residing in the intermembrane of mitochondria, was proved to be required in the regulation of cardiac hypertrophy. MICU1 was depressed in hypertrophic hearts, and *in vivo* and *in vitro* experimental data suggested that MICU1 ablation aggravated cardiac hypertrophy induced by Ang-II infusion, as evidenced by the worsened cardiac function, increased hypertrophic biomarkers, and cardiac histological alterations; in contrast, cardiac MICU1 supplementation ameliorated CM enlargement and blunted cardiac dysfunction (38). Utilizing transmission electron microscopy, researchers found that MICU1 knockdown was correlated with massive loss of mitochondrial membrane integrity, ambiguous cristae and myofilaments structures, besides, MICU1 deficiency aggravated the increase in mitochondrial mass, the suppression of MMP, and the depression of ATP contents in hypertrophic hearts. However, overexpression of MICU1 rescued mitochondrial morphological and functional abnormalities (38). Transient receptor potential channel, canonical 3(TRPC3), residing in mitochondria, also exerted regulatory roles in high salt-induced cardiac hypertrophy by mediating mitochondrial function. TRPC3 ablation antagonized cardiac hypertrophy induced by high salt intake, which was achieved by restoring the synthesis of ATP and the activity of complex I and II enzyme in mitochondria (39).

Taken together, mitochondrial Ca^2+^ in the heart plays central roles in stimulating ATP production and maintaining cardiac contractile activity in response to cardiac hypertrophy, thus strategies to main calcium homeostasis in the mitochondria are essential to block cardiac hypertrophic response. Despite great efforts, further investigations are still required to discover the cellular and molecular mechanisms underlying mitochondrial calcium homeostasis. Moreover, the real-time dynamic observation of the mitochondrial calcium alterations inside hypertrophic CMs may contribute to a better understanding of the link between mitochondrial Ca^2+^ and cardiac hypertrophy, and helps to develop novel therapeutic approaches to prevent or block pathological cardiac hypertrophy.

### Mitophagy

The autophagic clearance of damaged or unnecessary mitochondria in CMs is delicately mediated by a mitochondria-specific form of autophagy, named mitophagy (8). Mitophagy is regarded as a critical quality control mechanism to remove damaged or dysfunctional mitochondrial and maintain mitochondrial homeostasis in the heart. In mice subjected to TAC, mitophagy was transiently activated (3–7 days post-TAC), and downregulated below physiological levels thereafter. Importantly, depletion of Drp1 abolished mitophagy and accelerated cardiac hypertrophy and mitochondrial dysfunction induced by overload, however, restoration of mitophagy by Tat-Beclin 1 partially rescued mitophagy and attenuated mitochondrial dysfunction and cardiac hypertrophy during pressure overload (40). The essential regulatory roles of mitophagy in diabetic cardiomyopathy, which is characterized by cardiac hypertrophy, diastolic dysfunction and lipotoxicity, have been investigated. Results from immunofluorescence suggested that the localization of LC3 in mitochondria exhibited a significant increase in CMs isolated from mice receiving high-fat diet (HFD), indicating that HFD consumption notably upregulated mitophagy in the heart. Moreover, in Atg7- or Parkin-deficient HFD mice, cardiac hypertrophy, diastolic dysfunction and lipid accumulation deteriorated and mitophagy was dramatically impaired, suggesting that mitophagy may serve as an adaptive mechanism to protect against diabetic cardiomyopathy in an Atg7- or Parkin-dependent manner. Importantly, treatment with TAT-Beclin1, an activator of mitophagy, effectively restored mitochondrial dysfunction and prevented the development of diabetic cardiomyopathy (41). Another study has reported that PTENα, the first identified PTEN isoform, may function as an important mitochondrial quality controller, and the deficiency of PTENα may contribute to impaired mitochondrial clearance by mitophagy in the heart (42). The ablation of PTENα in mice led to an accumulation of structurally and functionally abnormal cardiac mitochondria; meanwhile, PTENα-depleted mice were more susceptible to ISO-induced cardiac injuries (42).

Collectively, in response to various pathological stresses, CMs selectively remove the dysfunctional mitochondria, reduce ROS production and restore cardiac function through the regulation of mitophagy. Exploring the roles of mitophagy in cardiac hypertrophy exists as an area of great interest.

### Enzymes in Mitochondria

Mitochondria contain thousands of different proteins, of which enzymes are an important component. In recent years, increasing lines of evidence support that mitochondrial enzymes, especially those involved in mitochondrial metabolism, have profound effects on cardiac hypertrophy and remodeling (43–48).

NADH: ubiquinone oxidoreductase core subunit S1 (Ndusf1), the prominent component of complex I, has gained particular attention in the regulation of cardiac hypertrophy. A recent study found that Ndusf1 was downregulated in TAC mouse models. Further gain- and loss-of-function analysis revealed that Ndusf1 deficiency deteriorated cardiac hypertrophic response (44). *In vitro* mechanistic exploration showed that the mitochondrial DNA content in CMs was significantly decreased after Ndusf1 deletion, consistently, loss of Ndusf1 dramatically elevated ROS production in CMs. Additionally, results from fluorescent staining illustrated that mitochondrial mass and mitochondrial membrane potential (MMP) were markedly repressed in CMs lack of Ndusf1. Ndusf1 overexpression, in turn, attenuated cardiac hypertrophy and mitochondrial dysfunction. A novel role of mitochondrial GTPases 1 (MTG1), which was known to participate in mitochondrial ribosome assembly and translation, in pathological cardiac hypertrophy has recently been revealed. MTG1 exhibited a significant increase in the heart tissue of both heart failure patients and mice subjected to AB surgery. Further loss- and gain-of-function studies showed that MTG1 depletion worsened AB-induced hypertrophy, whereas MTG1 overexpression mitigated cardiac hypertrophy and dysfunction after the onset of AB. The cardio-protective potentials of MTG1 deficiency were attributed to the improved activity of the mitochondrial respiratory chain complex and subsequently reduced ROS production (43). Mitochondrial Rho GTPase (Miro2), a well-known outer mitochondrial membrane protein mediating intracellular mitochondrial transport, was proved to be effective in the modulation of cardiac inter-mitochondrial communication during cardiac hypertrophy (45). In TAC-induced hypertrophic mouse hearts, downregulated Miro2 expression was detected, along with disrupted inter-mitochondrial communications. And promisingly, the Miro2 transgenic mice were protected from TAC-induced cardiac dysfunction, which was obtained by improving the communications between adjacent mitochondria and attenuating mitochondrial dysfunction (45). *In vivo* and *in vitro* experimental data also demonstrated that endonuclease G (Endog) loss-of-function may serve as a novel determinant of decompensated cardiac hypertrophy and impaired cardiac function. Importantly, in the hearts of Endog-deficient mice, there existed a dramatic increase in mitochondrial depletion and ROS generation, suggesting a previously unappreciated link between mitochondrial dysfunction and maladaptive cardiac hypertrophy (48). Mitochondrial NADP (+)-dependent isocitrate dehydrogenase (IDH2) has been well-known to mediate the balance of mitochondrial redox and help to maintain mitochondrial homeostasis. Notably, studies have also suggested that mitochondria isolated from the hearts of IDH2-deficient mice lost IDH2 activity and IDH2^−/−^ mice rapidly developed uncompensated HF, with increased hypertrophy and mitochondrial dysfunction, which was proved to be caused by the disturbance of the redox homeostasis (46). Glutaredoxin 2 (GRX2), a glutathione-dependent oxidoreductase in mitochondria, was essential in maintaining mitochondrial homeostasis and preserving cardiac structure and function. Based on the genetic and histopathological databases, researchers showed that relatively row expression of GRX2 indicated an increased incidence of human cardiac pathologies, including cardiac hypertrophy, fibrosis and infarct. Loss-of-function analysis also revealed that the absence of GRX2 was highly relevant with impaired mitochondrial dynamics, ultrastructure and energetics in both heart tissue and primary CMs (47).

In summary, the above results provide new insights into the possible molecular mechanisms underlying pathological cardiac hypertrophy, and further suggest that mitochondrial enzymes may represent potential therapeutic targets to alleviate cardiac hypertrophy and dysfunction.

### Sirtuin Family

Sirtuins (Sirt), consist of seven identified homologs (Sirt1-7), are a family of class III nicotinamide adenine dinucleotide (NAD+)-dependent deacetylase, among which Sirt3-5 are mainly resident in mitochondria. It has been well established that sirtuins exert protective effects on a variety of aging-related diseases by regulating some important cellular processes in the nucleus, cytoplasm as well as mitochondria to keep a balance of energy metabolism within the cell (49). In this section, we will give an overview of the critical roles of sirtuins, especially mitochondrial sirtuins, in pathological cardiac hypertrophy.

In mice with cardiac-specific *SIRT2* overexpression, Ang II-induced cardiac hypertrophy was repressed and cardiac dysfunction was rescued, whereas cardiac-specific depletion of Sirt2 dramatically aggravated hypertrophic response to Ang II stimulation. Furthermore, the authors showed that SIRT2 could directly bound to and interacted with LKB1, and the deacetylation of LKB1 participated in SIRT2-mediated suppression of stress-induced hypertrophic response (50). Sirt3, another important number of the sirtuin family, exhibited remarkable upregulation in the nuclear under ISO treatment. And adenovirus-mediated exogenous Sirt3 supplementation prevented PE- and ISO-induced hypertrophic response via deacetylating PARP-1 and inhibiting its activity (51). In HFD-induced cardiac hypertrophy and dysfunction, the absence of Sirt3 led to increased ROS generation and aggravated heart function compared with the WT mice, suggesting the significance of Sirt3 in preserving mitochondrial hemostasis and cardiac function during obesity-induced pathological hypertrophy (52). Sirt4, a sirtuin mainly present in mitochondria, plays an essential role in the progression of cardiac hypertrophy. Strictly, loss of Sirt4 protected the mice from Ang II-induced cardiac hypertrophy, whereas cardiac overexpression of Sirt4 accelerated cardiac maladaptation in response to Ang II treatment. Mechanistically, Sirt4 promoted the production of ROS by blunting Sirt3-mediated MnSOD activation, thereby enhancing cardiac decompensation upon Ang II infusion; and application of 4-benzoic acid to mimic MnSOD activity could eliminate Sirt4-mediated hypertrophic alterations (53). This research provided a novel link between mitochondrial oxidative stress and cardiac hypertrophy. Genetic ablation of Sirt5 accelerated cardiac dysfunction and increased mortality in the TAC mice. By performing MS-based proteomics and metabolomics analyses of heart tissues isolated from Sirt5-KO and WT mice, researchers observed an overall decrease in fatty acid oxidation, glucose oxidation and mitochondrial NAD+/NADH (54). Sirt7, the most recently identified sirtuin in mammals, has been proven to protect against TAC-induced cardiac hypertrophy. Additionally, SIRT6 served as a negative modulator of cardiac hypertrophy through interacting with c-Jun and suppressing IGF-Akt signaling (55). In cardiac-specific Sirt7-depletion mice, hypertrophic response was significantly augmented, as evidenced by increased heart weight/tibial length, larger CM cross-sectional area, and deteriorated heart function compared to WT mice receiving TAC operation, and further mechanism investigation indicated that The anti-hypertrophic roles of Sirt7 may be attributed to the deacetylation of GATA4 (56).

The beneficial effects of the sirtuin family on lipid metabolism, glucose oxidation, and ROS production in cardiac hypertrophy are at the pre-clinical level.

### Other Mitochondrial Proteins

By performing genome-wide exon array analysis in hypertrophic murine hearts induced by TAC surgery, Ito and colleagues successfully identified Mtus1A, one of the splice variants of mitochondrial tumor suppressor 1 (Mtus1), as a central modulator in hemodynamic stress-induced cardiac hypertrophy. In cardiac-specific Mtus1A transgenic mice, the hypertrophic response was remarkably mitigated, and *in vitro* experimental results revealed that Mtus1A overexpression alleviated the production of ROS and subsequent phosphorylation of ERK, contributing to a decrease in cell hypertrophy and protein synthesis (57). Translocase of inner membrane 50 (TIM50) was downregulated in the failing hearts, *in vivo* and *in vitro* loss- and gain-of-function analysis revealed that TIM50 acted as a novel repressor in cardiac hypertrophy. Overexpression of TIM50 significantly attenuated the increased cell size, oxidative stress and cellular apoptosis induced by Ang II (58). Miro1, a mitochondrial outer membrane protein, also played a central role in modulating mitochondrial dynamics and metabolism in cardiac hypertrophy. In NRCMs, PE stimulation provoked hypertrophic response as well as mitochondrial fission pattern, which were reversed when Miro1 was ablated (59). Inhibition of Dynamin-related protein1 (Drp1), a protein that regulates mitochondrial fission, with specific inhibitors exerted protective effects against ventricular hypertrophy induced by high salt intake, meanwhile, the ROS generation, as well as CaMKII expression were suppressed in Drp1-depleted rats (60). These studies illustrate that some proteins residing inside mitochondria are of great importance to maintain cardiac hemostasis, little changes in these proteins may trigger prominent mitochondrial dysfunction, imbalance in energy production, and pathological cardiac hypertrophy.

### MicroRNAs

Although the roles of microRNAs (miRs) in life activities have been widely investigated, there to date exist only a few studies revealing the functions of miRs in mitochondrial structure and function during cardiac hypertrophic response. A very recent study has identified miR-153-3p as a trigger of abnormal mitochondrial fission and hypertrophic response, mainly through the inhibition of Mfn1 translation, and both *in vivo* and *in vitro* experiments confirmed that the ablation of miR-153-3p could dramatically avoid mitochondrial fission as well as cardiac hypertrophy (61). Depletion of miR-106a almost entirely reversed AngII-induced hypertrophic phenotypes. Notably, Mfn2, a well-known mitochondrial fusion protein, was identified as a direct target for miR-106a. Silence of Mfn2 eliminated the anti-hypertrophic effects of miR-106a inhibitors, while Mfn2 overexpression abolished the pro-hypertrophic property of miR-106a. Additionally, miR-106a inhibition or Mfn2 overexpression significantly reversed mitochondrial cristae defects, depolarization of the mitochondrial membrane, and increased ROS generation caused by sustained cardiac overload (62). Similarly, miR-376b-3p attenuated mitochondrial fragmentation and hypertrophic response in noradrenaline-treated CMs, which was obtained by its direct interactions with mitochondrial fission factor (MFF) to modulate mitochondrial morphology and function (63). Another study has also shown that, in cultured CMs with PE treatment, miR-485-5p overexpression could diminish mitochondrial fragmentation and hypertrophic response by decreasing the level of MAPL expression and upregulating Mfn2 expression (64). In addition, miRs also appear to be effective during hypertrophic response. The latest research progress by Li's group suggested that miR-27b-3p deficiency remarkably reduced cardiac hypertrophy, fibrosis, and inflammation in both TAC and Angiotensin II (Ang II) perfusion-induced murine models; surprisingly, inhibition of endogenous miR-27b-3p could significantly enhance mitochondrial oxidative phosphorylation (OXPHOS) through activating PGC1α/β (65). Overexpression of miR-142-3p reversed MMP, mitochondrial density and effectively abolished hypertrophic response in CMs stimulated by Ang II, which was obtained by suppressing the expression of *SH2B1* (66). These results show that microRNAs are key regulators in mitochondrial function in the heart. The critical roles of microRNAs provide a new perspective on developing new diagnostic and therapeutic tools to block or reverse pathological cardiac hypertrophy.

### Regulators Outside Mitochondria

The mechanisms underlying mitochondrial dysfunction and cardiac hypertrophy are multifactorial and likely involve nuclear-encoded regulatory factors, residing outside the mitochondria, that directly or indirectly impact mitochondrial function and contribute to the progression of pathological cardiac hypertrophy. As such, manipulation of these critical regulators might fulfill a double role by suppressing mitochondrial abnormalities and improving cardiac function.

Nuclear factor of activated T cells, cytoplasmic 4 (NFATc4) overexpression aggravated phenylephrine (PE)-induced perturbations in mitochondrial genesis, membrane potential, and mitochondrial respiration; in contrast, NFATc4 deletion mitigated PE-induced mitochondrial dysfunction as well as cardiac hypertrophy (67). Mitochondrial dysfunction induced by Nlrp3 inflammasome also appears to play a regulatory role in Ang II-induced cardiomyopathy. The mitochondria in the hypertrophic heart tissue were swollen and fragmented, but these morphological alterations were significantly reversed in the NLRP3^−/−^ group. In addition to mitochondrial morphological alterations, cardiac hypertrophy also led to mitochondrial dysfunction, which was accompanied by insufficient mitochondrial biogenesis, damaged mtDNA, disordered intracellular ATP synthesis, and low levels of TFAM and PGC1a. Surprisingly, Nlrp3 depletion remarkably reversed the decreased expression of PGC1a and TFAM, abnormal mtDNA and ATP synthase in the hypertrophic heart caused by Ang II infusion (68). Polycystic kidney disease 2-like 1 (PKD2L1), known to serve as an important sour sensor in taste cells, played possible roles in cardiac hypertrophy. *In vitro* and *in vivo* experiments showed that hypertrophic stress (including high salt intake and Ang II infusion) significantly increased the expression of PKD2L1 in mitochondria. PKD2L1-deficient mice exhibited aggravated pathological cardiac hypertrophy caused by a high salt diet, at the same time, less O2 consumption, reduced heat production and a decreased respiratory exchange ratio were also observed in mice with PKD2L1 mutation. Further mechanical investigation illustrated that PKD2L1 was capable of controlling the deteriorated mitochondrial Ca^2+^ overload mediated by NCX1, via restricting p300-mediated histone acetylation on the NCX1 promoter (69). The Grb2-associated binder 1 (Gab1), a key mediator of growth factor receptor signaling, also participated in the regulation of cardiac hypertrophy induced by pressure overload. The levels of Gab1 were diminished in both human and mouse DCM hearts. Cardiac-specific Gab1 knockout mice rapidly developed severe DCM and heart failure after the onset of cardiac hemodynamic stress, furthermore, gene microarray analysis revealed the upregulation of several pro-apoptotic genes and downregulation of some key anti-apoptotic genes. Further analysis of mitochondrial function and caspase activity demonstrated that under hemodynamic stress conditions, Gab1 deficiency caused severe damage to both mitochondrial ultrastructure and function and triggered massive activation of caspase, consequently leading to CM apoptosis and heart failure (70).

**Figure 1:**
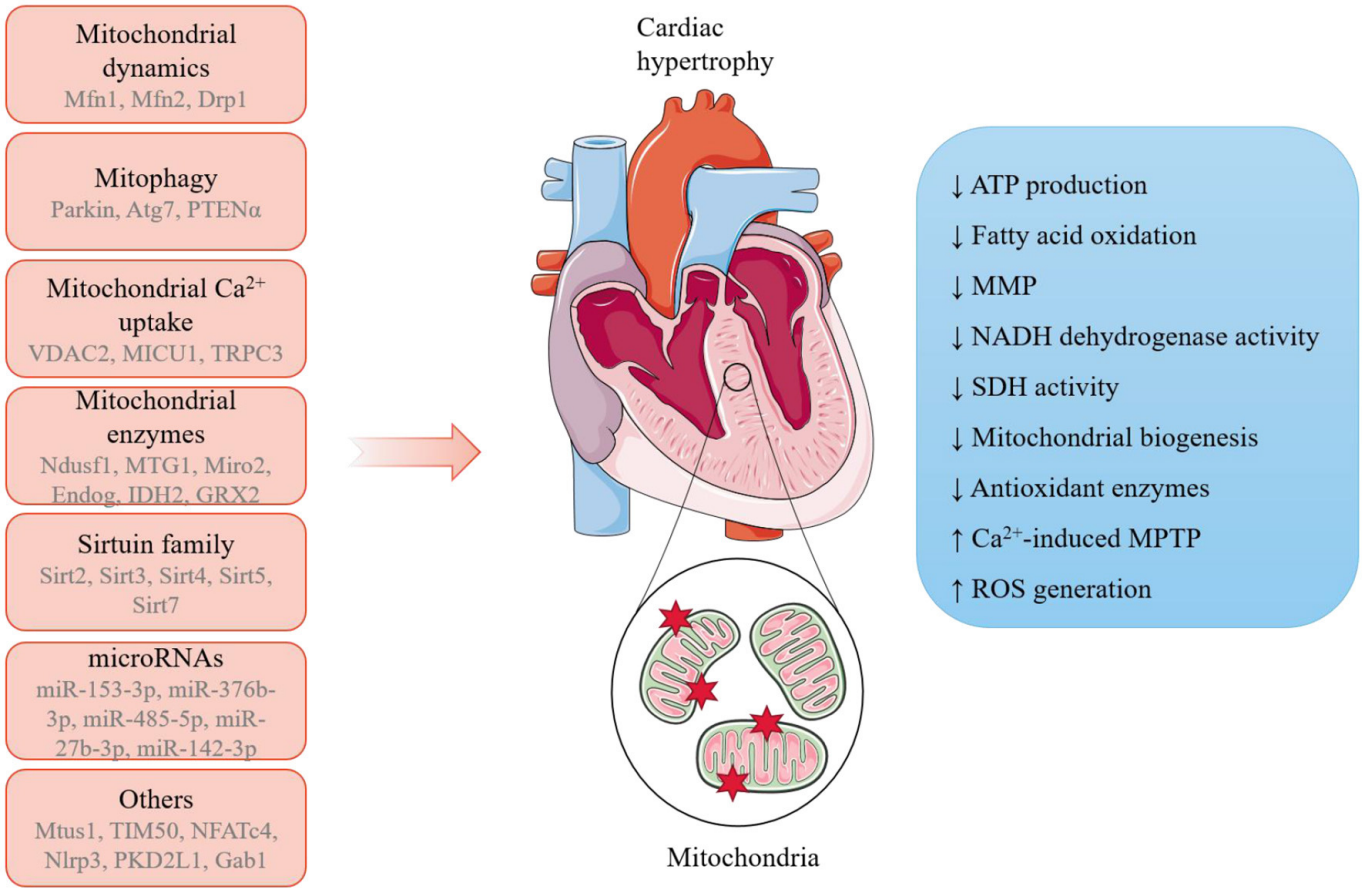
A summary of the potential mechanisms linking mitochondria and pathological cardiac hypertrophy. A range of mitochondria-dependent and -independent regulators have been reported to participate in the initiation and progression of pathological cardiac hypertrophy, during which mitochondria undergo remarkable morphological and functions alterations.

In a word, these latest studies provide new mechanistic insights into the link between mitochondria and cardiac hypertrophy (Figure 1). In-depth exploration of mitochondria-mediated cellular and molecular regulation would provide a better understanding of the etiology and progression of cardiac hypertrophy and support the discovery of novel biomarkers in diseases and therapeutic targets to fight against cardiomyopathies.

## Potential Therapeutic Approaches Targeting Mitochondria to Prevent or Reverse Cardiac Hypertrophy

### Phytochemicals

An attractive pharmacological tool for the treatment of cardiac hypertrophy is the traditional phytochemicals. A growing number of studies have proved that phytochemicals are capable of mitigating cardiac hypertrophy in cell and animal models via modulating mitochondrial function and maintaining mitochondrial hemostasis. Wei's recent findings suggest that T89, a traditional Chinese medicine, may be a potential therapeutic approach to treat ISO-induced cardiac hypertrophy, and the underlying mechanisms mainly lay on the regulation of metabolic pathways (71). By performing quantitative proteomics, the group revealed that T_89_ could inhibit glycolysis, accelerate fatty acid oxidation and restore mitochondrial OXPHOS, thereby attenuating energy metabolism disorders and alleviating ISO-induced cardiac injury. Bawei Chenxiang Wan (BCW), another well-known traditional Chinese medicine, was approved to be effective in ISO-induced rat hypertrophic models. Moreover, a number of mitochondrial DNA-encoded genes, such as *ND1, Cyt b, ATP 5*β, and *mt-coI* were downregulated in ISO-treated rats, but restored when administrated with BCW, indicating that the anti-hypertrophic roles of BCW were obtained by modulating mitochondria function and oxidative phosphorylation (72). Mitophagy may exist as another important target. Berberine has recently been verified to improve heart function in cardiac hypertrophy murine models, which was exactly achieved by the activation of mitophagy via the PINK1/Parkin/Ubiquitination signaling pathway (73). Similarly, the pretreatment of baicalein significantly attenuated cardiac hypertrophy and restored cardiac function in experimental *in vivo/vitro* models by activating mitophagy (74). As a natural bisphenol compound extracted from the magnolia bark, Honokiol (HKL) exerted cardio-protective properties in pathological cardiac hypertrophy. Interestingly, HKL administration substantially decreased the acetylation levels of mitochondrial proteins via directly binding to and activating mitochondrial SIRT3 (75). Similarly, Emodin, as the main component of rhubarb, could effectively block ISO- and TAC-induced cardiac hypertrophy through Sirt3-dependent mitochondrial protection (76).

### Antioxidants

In view of the extensive ROS production and severe oxidative stress after the onset of cardiac overload, treatment with antioxidants may represent an efficient approach to attenuate mitochondrial dysfunction and block cardiac hypertrophy. Recent research has found that the application of mitoTEMPO (an antioxidant targeting mitochondrial ROS) or resveratrol (a sirtuin agonist) fulfilled a protective role against nicotine-induced cardiac hypertrophy, fibrosis and inflammation and dysfunction in rats (77). Alpha-lipoic acid (α-LA), another well-known antioxidant, was also proved to protect against TAC-induced adverse cardiac hypertrophy, cardiac dysfunction and mitochondrial dysfunction via ALDH2-dependent activation of a novel mitophagy receptor protein FUNDC1 (78). Similarly, pretreatment with NaHS abolished hypertrophic response, mitochondrial ultrastructure impairment, mitochondrial depletion and oxidative stress during cardiac pressure overload in a Sirt3-dependent way (79). Additionally, lactoferrin seemed to be an effective agent against pathological cardiac hypertrophy in aged mice. After lactoferrin intake, the aged mice showed remarkable improvement of cardiac hypertrophy and dysfunction, which were partly attributed to mitochondrial dynamic rearrangement and the control of mitochondrial-lysosomal axis-related detrimental regulators (80). Interestingly, researchers have recently illustrated the relationship between aerobic exercise and hypertrophy, and they illustrated that in the mice persisting in aerobic exercise, cardiac hypertrophy and dysfunction were greatly improved. Mechanistically, mitochondria from the aerobic exercise group exhibited decreased excessive mitochondrial fission and mitochondrial autophagy, thus restoring mitochondrial ultrastructure and function in response to hypertrophic insults (81).

### Other Mitochondria-Targeted Molecules

A very recent study has found that external supplementation of alpha-ketoglutarate (AKG), an intermediate metabolite of the tricarboxylic acid cycle, effectively suppressed transverse aortic constriction (TAC)-induced cardiac hypertrophy and improved heart function (82). The cardio-protective roles of AKG may be achieved by promoting mitophagy, which helped to remove damaged mitochondria and reduce ROS production (82). Allyl Methyl Sulfide (AMS) intervention also exerted protective effects on TAC-induced cardiac hypertrophy. Mechanistically, PCR array experiments showed that AMS could improve mitochondrial energy metabolism-related genes compared to vehicle control; AMS pre-treatment also restored mitochondrial oxygen consumption rate, MMP and ROS production in CMs stimulated by ISO, mitochondria fusion and fission were also balanced after AMS intervention (83). Pharmacological intervention targeting ATP-sensitive K^+^ channels in mitochondria (mitoKATP) may act as a potential therapeutic approach to alleviate cardiac hypertrophy. Treatment with diazoxide in ISO-induced cardiac hypertrophy mice to open mitoKATP significantly improved MnSOD activity and decreased H_2_O_2_ production, and it went the opposite when treated with 5-hydroxydecanoate or glibenclamide (mitoKATP blockers), indicating that mitoKATP opening seems promising to block oxidative stress and ameliorate cardiac hypertrophy (84). Interestingly, the administration of choline protected against abdominal aorta banding (AAB) and Ang II-induced cardiac hypertrophy and dysfunction. In hypertrophic groups pre-treated with choline, the mito-nuclear protein imbalance was reversed and the unfolded protein response of mitochondria (UPRmt) was greatly activated, thus mitochondrial ultrastructure and function were restored to normal (21).

## Conclusions

Mitochondria are the prominent organelle for the energy supply of CMs, and a large number of researches have demonstrated that mitochondria are highly susceptible to pathological stimuli, including sustained and repeated cardiac overload (5, 16). Pathological cardiac hypertrophy results in functionally and structurally damaged mitochondria, and in turn, the abnormal mitochondria may also mediate cardiac hypertrophic response via regulatory pathways inside and outside mitochondria. It is widely accepted that balanced regulation of mitochondrial energy supply and the cardiac energy consumption is extremely delicate. Thus, efficient cardiac therapies targeting mitochondrial lesions and dysfunction may constitute a new strategy to inhibit the high susceptibility to pro-hypertrophic stimuli and attenuate pathological cardiac hypertrophy (Table 2).

**Table 2:** A summary of therapeutic approaches targeting mitochondria to treat cardiac hypertrophy.

**Potential therapy**	**Models**	**Observations**	**Mechanisms**
T89 (71)	ISO-induced rat cardiac hypertrophy	Attenuated cardiac dysfunction and energy metabolism disorders	inhibiting glycolysis, accelerating fatty acid oxidation and restoring mitochondrial OXPHOS
Bawei Chenxiang Wan (72)	ISO-induced rat hypertrophic models	Alleviated cardiac injury and restored mitochondrial DNA-encoded genes	modulating mitochondria function and oxidative phosphorylation
Berberine (73)	TAC-induced murine cardiac hypertrophy	Improved heart function	Activating mitophagy via the PINK1/Parkin/Ubiquitination signaling pathway
Baicalein (74)	ISO-induced murine cardiac hypertrophy	Attenuated cardiac hypertrophy and cardiac function	Activating mitophagy
Honokiol (75)	TAC/ISO-induced murine cardiac hypertrophy	Attenuated cardiac hypertrophy and cardiac function	Decreasing mitochondrial protein acetylation via regulating SIRT3
Emodin (76)	TAC/ISO-induced murine cardiac hypertrophy	Improved ISO- and TAC-induced cardiac hypertrophy	Regulating SIRT3 signaling pathway
Alpha-lipoic acid (78)	TAC-induced murine cardiac hypertrophy	Improved cardiac hypertrophy and function	ALDH2-dependent activation of a novel mitophagy receptor protein FUNDC1
NaHS (79)	TAC-induced murine cardiac hypertrophy	Improved hypertrophic response and mitochondrial function	In a Sirt3-dependent way
Lactoferrin (80)	Aging-related cardiac hypertrophy	Improved cardiac hypertrophy and function	Improving mitochondrial dynamics and mitochondrial-lysosomal axis
aerobic exercise (81)	TAC-induced murine cardiac hypertrophy	Improved cardiac hypertrophy and function	Decreasing excessive mitochondrial fission and mitochondrial autophagy
alpha-ketoglutarate (82)	TAC-induced murine cardiac hypertrophy	Improved cardiac hypertrophy and function	Promoting mitophagy
Allyl Methyl Sulfide (83)	TAC-induced rat cardiac hypertrophy	Improved cardiac hypertrophy and function	Improving mitochondrial energy metabolism
Diazoxide (84)	ISO-induced murine cardiac hypertrophy	Attenuated cardiac hypertrophy	Opening mitoKATP, improving MnSOD activity and decreasing H_2_O_2_ production

Despite great progress has been made in elucidating the potential regulatory mechanisms underlying cardiac hypertrophy, there are still some issues that remain to be solved. Growing studies have provided evidence that mitochondria exert the regulatory roles on cardiac hypertrophy via countless modulators or signaling pathways, meanwhile, one particular regulator may have multiple effects related or unrelated to hypertrophy, thus identifying the prioritized mechanisms altered and essential in humans is clinically significant and should be highly valued. Selectively suppressing the specific signaling mechanisms is of significant importance in enhancing heart function in patients suffering from cardiac hypertrophy. Moreover, in recent years, increasing studies have revealed the potential protective roles of some agents against cardiac hypertrophy via impacting mitochondrial function, however, these possibilities are mainly verified in animal experiments but are poorly characterized in clinical trials. Future work should also be directed on improving their specificity and efficacy as well as avoiding the potential cytotoxicity.

Another vagueness lies on the causal link between mitochondrial abnormalities and cardiac hypertrophy. It seems that during the hypertrophic response, notable mitochondrial damage was detected, in addition, mitochondria-mediated signaling pathways contribute to the initiation and progression of pathological hypertrophy. It is hard to determine whether structural and functional alterations in mitochondria are the primary cause or secondary effects in pathological cardiac hypertrophy. A detailed time-course study in hypertrophy models may help to explain the issues.

## Author Contributions

DY, DF, and Q-ZT contributed to the conception and design of the review. The draft of the manuscript was written by DY and H-QL, F-YL, ZG, PA, and M-YW performed the literature search. ZY, DF, and Q-ZT critically revised the manuscript. All authors contributed to the article and approved the submitted version.

## Funding

This work was supported by grants from the National Natural Science Foundation of China (81900219, 81800216). The funds received are used for open access publication fees.

## Conflict of Interest

The authors declare that the research was conducted in the absence of any commercial or financial relationships that could be construed as a potential conflict of interest.

## Publisher's Note

All claims expressed in this article are solely those of the authors and do not necessarily represent those of their affiliated organizations, or those of the publisher, the editors and the reviewers. Any product that may be evaluated in this article, or claim that may be made by its manufacturer, is not guaranteed or endorsed by the publisher.

## Abbreviations

CMs: Cardiomyocytes
NRCMs: normal neonatal rat cardiomyocytes
NF: non-failing
HFrEF: reduced ejection fraction
MMP: mitochondrial membrane potential
SDH: succinate dehydrogenase
Mfn: mitofusin
VDAC2: Voltage-dependent anion channel 2
DCM: dilated cardiomyopathy
TEM: Transmission electron microscopy
MICU1: Mitochondrial calcium uptake 1
TRPC3: Transient receptor potential channel canonical 3
ISO: isoproterenol
Sirt: Sirtuins
Mtus1: mitochondrial tumor suppressor 1.

## References

[1] Shimizu I Minamino T. Physiological and pathological cardiac hypertrophy. J Mol Cell Cardiol. (2016) 97:245–62. 10.1016/j.yjmcc.2016.06.001 27262674

[2] Gibb AA Epstein PN Uchida S Zheng Y McNally LA Obal D. Exercise-induced changes in glucose metabolism promote physiological cardiac growth. Circulation. (2017) 136:2144–57. 10.1161/CIRCULATIONAHA.117.028274 28860122 PMC5704654

[3] Yang D Liu HQ Liu FY Tang N Guo Z Ma SQ. The roles of noncardiomyocytes in cardiac remodeling. Int J Biol Sci. (2020) 16:2414–29. 10.7150/ijbs.47180 32760209 PMC7378633

[4] Yang D Liu HQ Liu FY Tang N Guo Z Ma SQ. Critical roles of macrophages in pressure overload-induced cardiac remodeling. J Mol Med (Berl). (2021) 99:33–46. 10.1007/s00109-020-02002-w 33130927

[5] Nakamura M Sadoshima J. Mechanisms of physiological and pathological cardiac hypertrophy. Nat Rev Cardiol. (2018) 15:387–407. 10.1038/s41569-018-0007-y 29674714

[6] Braunwald E. The war against heart failure: the Lancet lecture. Lancet. (2015) 385:812–24. 10.1016/S0140-6736(14)61889-4 25467564

[7] Barth E Stämmler G Speiser B Schaper J. Ultrastructural quantitation of mitochondria and myofilaments in cardiac muscle from 10 different animal species including man. J Mol Cell Cardiol. (1992) 24:669–81. 10.1016/0022-2828(92)93381-S 1404407

[8] Tong M Sadoshima J. Mitochondrial autophagy in cardiomyopathy. Curr Opin Genet Dev. (2016) 38:8–15. 10.1016/j.gde.2016.02.006 27003723 PMC5028232

[9] Ramachandra CJA Hernandez-Resendiz S Crespo-Avilan GE Lin YH Hausenloy DJ. Mitochondria in acute myocardial infarction and cardioprotection. EBioMed. (2020) 57:102884. 10.1016/j.ebiom.2020.102884 32653860 PMC7355051

[10] Picca A Mankowski RT Burman JL Donisi L Kim JS Marzetti E. Mitochondrial quality control mechanisms as molecular targets in cardiac ageing. Nat Rev Cardiol. (2018) 15:543–54. 10.1038/s41569-018-0059-z 30042431 PMC6283278

[11] Marin W Marin D Ao X Liu Y. Mitochondria as a therapeutic target for cardiac ischemia-reperfusion injury (Review). Int J Mol Med. (2021) 47:485–99. 10.3892/ijmm.2020.4823 33416090 PMC7797474

[12] Peng W Cai G Xia Y Chen J Wu P Wang Z. Mitochondrial Dysfunction in Atherosclerosis. DNA Cell Biol. (2019) 38:597–606. 10.1089/dna.2018.4552 31095428

[13] Abel ED Doenst T. Mitochondrial adaptations to physiological vs. pathological cardiac hypertrophy. Cardiovasc Res. (2011) 90:234–42. 10.1093/cvr/cvr015 21257612 PMC3115280

[14] Dai DF Johnson SC Villarin JJ Chin MT Nieves-Cintrón M Chen T. Mitochondrial oxidative stress mediates angiotensin II-induced cardiac hypertrophy and Galphaq overexpression-induced heart failure. Circ Res. (2011) 108:837–46. 10.1161/CIRCRESAHA.110.232306 21311045 PMC3785241

[15] Wüst RC de Vries HJ Wintjes LT Rodenburg RJ Niessen HW Stienen GJ. Mitochondrial complex I dysfunction and altered NAD(P)H kinetics in rat myocardium in cardiac right ventricular hypertrophy and failure. Cardiovasc Res. (2016) 111:362–72. 10.1093/cvr/cvw176 27402402

[16] Facundo H Brainard RE Caldas FRL Lucas AMB. Mitochondria and cardiac hypertrophy. Adv Exp Med Biol. (2017) 982:203–26. 10.1007/978-3-319-55330-6_11 28551789

[17] Heine KB Hood WR. Mitochondrial behaviour, morphology, and animal performance. Biol Rev Camb Philos Soc. (2020) 95:730–7. 10.1111/brv.12584 32022456

[18] Dorn GW 2nd Vega RB Kelly DP. Mitochondrial biogenesis and dynamics in the developing and diseased heart. Genes Dev. (2015) 29:1981–91. 10.1101/gad.269894.115 26443844 PMC4604339

[19] Song M Franco A Fleischer JA Zhang L Dorn GW 2nd. Abrogating mitochondrial dynamics in mouse hearts accelerates mitochondrial senescence. Cell Metabol. (2017) 26:872–83.e5. 10.1016/j.cmet.2017.09.023 29107503 PMC5718956

[20] Gao S Li G Shao Y Wei Z Huang S Qi F. FABP5 deficiency impairs mitochondrial function and aggravates pathological cardiac remodeling and dysfunction. Cardiovasc Toxicol. (2021) 21:619–29. 10.1007/s12012-021-09653-2 33929718

[21] Xu M Xue RQ Lu Y Yong SY Wu Q Cui YL. Choline ameliorates cardiac hypertrophy by regulating metabolic remodelling and UPRmt through SIRT3-AMPK pathway. Cardiovasc Res. (2019) 115:530–45. 10.1093/cvr/cvy217 30165480

[22] Chen L Gong Q Stice JP Knowlton AA. Mitochondrial OPA1, apoptosis, and heart failure. Cardiovasc Res. (2009) 84:91–9. 10.1093/cvr/cvp181 19493956 PMC2741347

[23] Xue RQ Zhao M Wu Q Yang S Cui YL Yu XJ. Regulation of mitochondrial cristae remodelling by acetylcholine alleviates palmitate-induced cardiomyocyte hypertrophy. Free Radic Biol Med. (2019) 145:103–17. 10.1016/j.freeradbiomed.2019.09.025 31553938

[24] Tigchelaar W de Jong AM Bloks VW van Gilst WH de Boer RA Silljé HH. Hypertrophy induced KIF5B controls mitochondrial localization and function in neonatal rat cardiomyocytes. J Mol Cell Cardiol. (2016) 97:70–81. 10.1016/j.yjmcc.2016.04.005 27094714

[25] Luo X Yin J Dwyer D Yamawaki T Zhou H Ge H. Chamber-enriched gene expression profiles in failing human hearts with reduced ejection fraction. Sci Rep. (2021) 11:11839. 10.1038/s41598-021-91214-2 34088950 PMC8178406

[26] Zhang L Jaswal JS Ussher JR Sankaralingam S Wagg C Zaugg M. Cardiac insulin-resistance and decreased mitochondrial energy production precede the development of systolic heart failure after pressure-overload hypertrophy. Circ Heart Fail. (2013) 6:1039–48. 10.1161/CIRCHEARTFAILURE.112.000228 23861485

[27] Nomura S Satoh M Fujita T Higo T Sumida T Ko T. Cardiomyocyte gene programs encoding morphological and functional signatures in cardiac hypertrophy and failure. Nat Commun. (2018) 9:4435. 10.1038/s41467-018-06639-7 30375404 PMC6207673

[28] Su H Pistolozzi M Shi X Sun X Tan W. Alterations in NO/ROS ratio and expression of Trx1 and Prdx2 in isoproterenol-induced cardiac hypertrophy. Acta Biochim Biophys Sin (Shanghai). (2017) 49:1022–8. 10.1093/abbs/gmx102 29036266

[29] Müller OJ Heckmann MB Ding L Rapti K Rangrez AY Gerken T. Comprehensive plasma and tissue profiling reveals systemic metabolic alterations in cardiac hypertrophy and failure. Cardiovasc Res. (2019) 115:1296–305. 10.1093/cvr/cvy274 30418544

[30] Lee H Yoon Y. Mitochondrial fission and fusion. Biochem Soc Trans. (2016) 44:1725–35. 10.1042/BST20160129 27913683

[31] Chen Y Dorn GW 2nd. PINK1-phosphorylated mitofusin 2 is a Parkin receptor for culling damaged mitochondria. Science. (2013) 340:471–5. 10.1126/science.1231031 23620051 PMC3774525

[32] Song M Mihara K Chen Y Scorrano L Dorn GW 2nd. Mitochondrial fission and fusion factors reciprocally orchestrate mitophagic culling in mouse hearts and cultured fibroblasts. Cell Metabol. (2015) 21:273–86. 10.1016/j.cmet.2014.12.011 25600785 PMC4318753

[33] Ikeda Y Shirakabe A Maejima Y Zhai P Sciarretta S Toli J. Endogenous Drp1 mediates mitochondrial autophagy and protects the heart against energy stress. Circ Res. (2015) 116:264–78. 10.1161/CIRCRESAHA.116.303356 25332205

[34] Chen Y Liu Y Dorn GW 2nd. Mitochondrial fusion is essential for organelle function and cardiac homeostasis. Circ Res. (2011) 109:1327–31. 10.1161/CIRCRESAHA.111.258723 22052916 PMC3237902

[35] Luczak ED Wu Y Granger JM Joiner MA Wilson NR Gupta A. Mitochondrial CaMKII causes adverse metabolic reprogramming and dilated cardiomyopathy. Nat Commun. (2020) 11:4416. 10.1038/s41467-020-18165-6 32887881 PMC7473864

[36] Dia M Gomez L Thibault H Tessier N Leon C Chouabe C. Reduced reticulum-mitochondria Ca(2+) transfer is an early and reversible trigger of mitochondrial dysfunctions in diabetic cardiomyopathy. Basic Res Cardiol. (2020) 115:74. 10.1007/s00395-020-00835-7 33258101 PMC7704523

[37] Shankar TS Ramadurai DKA Steinhorst K Sommakia S Badolia R Thodou Krokidi A. Cardiac-specific deletion of voltage dependent anion channel 2 leads to dilated cardiomyopathy by altering calcium homeostasis. Nat Commun. (2021) 12:4583. 10.1038/s41467-021-24869-0 34321484 PMC8319341

[38] Yang Y Du J Xu R Shen Y Yang D Li D. Melatonin alleviates angiotensin-II-induced cardiac hypertrophy via activating MICU1 pathway. Aging. (2020) 13:493–515. 10.18632/aging.202159 33259334 PMC7834983

[39] Ma T Lin S Wang B Wang Q Xia W Zhang H. TRPC3 deficiency attenuates high salt-induced cardiac hypertrophy by alleviating cardiac mitochondrial dysfunction. Biochem Biophys Res Commun. (2019) 519:674–81. 10.1016/j.bbrc.2019.09.018 31543348

[40] Shirakabe A Zhai P Ikeda Y Saito T Maejima Y Hsu CP. Drp1-dependent mitochondrial autophagy plays a protective role against pressure overload-induced mitochondrial dysfunction and heart failure. Circulation. (2016) 133:1249–63. 10.1161/CIRCULATIONAHA.115.020502 26915633 PMC4811679

[41] Tong M Saito T Zhai P Oka SI Mizushima W Nakamura M. Mitophagy is essential for maintaining cardiac function during high fat diet-induced diabetic cardiomyopathy. Circ Res. (2019) 124:1360–71. 10.1161/CIRCRESAHA.118.314607 30786833 PMC6483841

[42] Li G Yang J Yang C Zhu M Jin Y McNutt MA. PTENα regulates mitophagy and maintains mitochondrial quality control. Autophagy. (2018) 14:1742–60. 10.1080/15548627.2018.1489477 29969932 PMC6135630

[43] Xu D Zhao Y Weng X Lu Y Li W Tang K. Novel role of mitochondrial GTPases 1 in pathological cardiac hypertrophy. J Mol Cell Cardiol. (2019) 128:105–16. 10.1016/j.yjmcc.2019.01.025 30707992

[44] Zou R Tao J Qiu J Shi W Zou M Chen W. Ndufs1 deficiency aggravates the mitochondrial membrane potential dysfunction in pressure overload-induced myocardial hypertrophy. Oxid Med Cell Longev. (2021) 2021:5545261. 10.1155/2021/5545261 33763166 PMC7952157

[45] Cao Y Xu C Ye J He Q Zhang X Jia S. Miro2 regulates inter-mitochondrial communication in the heart and protects against TAC-induced cardiac dysfunction. Circ Res. (2019) 125:728–43. 10.1161/CIRCRESAHA.119.315432 31455181

[46] Ku HJ Ahn Y Lee JH Park KM Park JW. IDH2 deficiency promotes mitochondrial dysfunction and cardiac hypertrophy in mice. Free Radic Biol Med. (2015) 80:84–92. 10.1016/j.freeradbiomed.2014.12.018 25557279

[47] Kanaan GN Ichim B Gharibeh L Maharsy W Patten DA Xuan JY. Glutaredoxin-2 controls cardiac mitochondrial dynamics and energetics in mice, and protects against human cardiac pathologies. Redox Biol. (2018) 14:509–21. 10.1016/j.redox.2017.10.019 29101900 PMC5675898

[48] McDermott-Roe C Ye J Ahmed R Sun XM Serafín A Ware J. Endonuclease G is a novel determinant of cardiac hypertrophy and mitochondrial function. Nature. (2011) 478:114–8. 10.1038/nature10490 21979051 PMC3189541

[49] Winnik S Auwerx J Sinclair DA Matter CM. Protective effects of sirtuins in cardiovascular diseases: from bench to bedside. Eur Heart J. (2015) 36:3404–12. 10.1093/eurheartj/ehv290 26112889 PMC4685177

[50] Tang X Chen XF Wang NY Wang XM Liang ST Zheng W. SIRT2 Acts as a cardioprotective deacetylase in pathological cardiac hypertrophy. Circulation. (2017) 136:2051–67. 10.1161/CIRCULATIONAHA.117.028728 28947430 PMC5698109

[51] Feng X Wang Y Chen W Xu S Li L Geng Y. SIRT3 inhibits cardiac hypertrophy by regulating PARP-1 activity. Aging. (2020) 12:4178–92. 10.18632/aging.102862 32139662 PMC7093179

[52] Zeng H Vaka VR He X Booz GW Chen JX. High-fat diet induces cardiac remodelling and dysfunction: assessment of the role played by SIRT3 loss. J Cell Mol Med. (2015) 19:1847–56. 10.1111/jcmm.12556 25782072 PMC4549035

[53] Luo YX Tang X An XZ Xie XM Chen XF Zhao X. SIRT4 accelerates Ang II-induced pathological cardiac hypertrophy by inhibiting manganese superoxide dismutase activity. Eur Heart J. (2017) 38:1389–98. 10.1093/eurheartj/ehw138 27099261

[54] Hershberger KA Abraham DM Martin AS Mao L Liu J Gu H. Sirtuin 5 is required for mouse survival in response to cardiac pressure overload. J Biol Chem. (2017) 292:19767–81. 10.1074/jbc.M117.809897 28972174 PMC5712617

[55] Sundaresan NR Vasudevan P Zhong L Kim G Samant S Parekh V. The sirtuin SIRT6 blocks IGF-Akt signaling and development of cardiac hypertrophy by targeting c-Jun. Nat Med. (2012) 18:1643–50. 10.1038/nm.2961 23086477 PMC4401084

[56] Yamamura S Izumiya Y Araki S Nakamura T Kimura Y Hanatani S. Cardiomyocyte Sirt (Sirtuin) 7 Ameliorates Stress-Induced Cardiac Hypertrophy by Interacting With and Deacetylating GATA4. Hypertension. (2020) 75:98–108. 10.1161/HYPERTENSIONAHA.119.13357 31735083

[57] Ito S Asakura M Liao Y Min KD Takahashi A Shindo K. Identification of the Mtus1 splice variant as a novel inhibitory factor against cardiac hypertrophy. J Am Heart Assoc. (2016) 5:e003521. 10.1161/JAHA.116.003521 27385424 PMC5015389

[58] Tang K Zhao Y Li H Zhu M Li W Liu W. Translocase of inner membrane 50 functions as a novel protective regulator of pathological cardiac hypertrophy. J Am Heart Assoc. (2017) 6:e004346. 10.1161/JAHA.116.004346 28432072 PMC5532988

[59] Conejeros C Parra V Sanchez G Pedrozo Z Olmedo I. Miro1 as a novel regulator of hypertrophy in neonatal rat cardiomyocytes. J Mol Cell Cardiol. (2020) 141:65–9. 10.1016/j.yjmcc.2020.03.014 32234389

[60] Hasan P Saotome M Ikoma T Iguchi K Kawasaki H Iwashita T. Mitochondrial fission protein, dynamin-related protein 1, contributes to the promotion of hypertensive cardiac hypertrophy and fibrosis in Dahl-salt sensitive rats. J Mol Cell Cardiol. (2018) 121:103–6. 10.1016/j.yjmcc.2018.07.004 29981304

[61] Wang T Zhai M Xu S Ponnusamy M Huang Y Liu CY. NFATc3-dependent expression of miR-153-3p promotes mitochondrial fragmentation in cardiac hypertrophy by impairing mitofusin-1 expression. Theranostics. (2020) 10:553–66. 10.7150/thno.37181 31903137 PMC6929994

[62] Guan X Wang L Liu Z Guo X Jiang Y Lu Y. miR-106a promotes cardiac hypertrophy by targeting mitofusin 2. J Mol Cell Cardiol. (2016) 99:207–17. 10.1016/j.yjmcc.2016.08.016 27565029

[63] Sun YL Li SH Yang L Wang Y. miR-376b-3p attenuates mitochondrial fission and cardiac hypertrophy by targeting mitochondrial fission factor. Clin Exp Pharmacol Physiol. (2018) 45:779–87. 10.1111/1440-1681.12938 29570827

[64] Zhao Y Ponnusamy M Liu C Tian J Dong Y Gao J. MiR-485-5p modulates mitochondrial fission through targeting mitochondrial anchored protein ligase in cardiac hypertrophy. Mol Basis Dis. (2017) 1863:2871–81. 10.1016/j.bbadis.2017.07.034 28782654

[65] Li G Shao Y Guo HC Zhi Y Qiao B Ma K. MicroRNA-27b-3p downregulates FGF1 and aggravates pathological cardiac remodelling. Cardiovasc Res. (2021) cvab248. 10.1093/cvr/cvab248 34358309 PMC9302889

[66] Liu BL Cheng M Hu S Wang S Wang L Tu X. Overexpression of miR-142-3p improves mitochondrial function in cardiac hypertrophy. Biomed Pharmacother. (2018) 108:1347–56. 10.1016/j.biopha.2018.09.146 30372837

[67] Liu X Gao S Gao H Jiang X Wei Q. Mitochondrial disruption is involved in the effect of nuclear factor of activated t cells, cytoplasmic 4 on aggravating cardiomyocyte hypertrophy. J Cardiovasc Pharmacol. (2021) 77:557–69. 10.1097/FJC.0000000000000986 33951694

[68] Chen Y Zeng M Zhang Y Guo H Ding W Sun T. Nlrp3 deficiency alleviates angiotensin ii-induced cardiomyopathy by inhibiting mitochondrial dysfunction. Oxid Med Cell Longev. (2021) 2021:6679100. 10.1155/2021/6679100 33628380 PMC7884178

[69] Lu Z Cui Y Wei X Gao P Zhang H Wei X. Deficiency of PKD2L1 (TRPP3) exacerbates pathological cardiac hypertrophy by augmenting NCX1-mediated mitochondrial calcium overload. Cell Rep. (2018) 24:1639–52. 10.1016/j.celrep.2018.07.022 30089272

[70] Zhao J Yin M Deng H Jin FQ Xu S Lu Y. Cardiac Gab1 deletion leads to dilated cardiomyopathy associated with mitochondrial damage and cardiomyocyte apoptosis. Cell Death Differ. (2016) 23:695–706. 10.1038/cdd.2015.143 26517531 PMC4986641

[71] Wei XH Guo X Pan CS Li H Cui YC Yan L. Quantitative proteomics reveal that metabolic improvement contributes to the cardioprotective effect of T(89) on isoproterenol-induced cardiac injury. Front Physiol. (2021) 12:653349. 10.3389/fphys.2021.653349 34262469 PMC8273540

[72] Zhang X Zhang Z Wang P Han Y Liu L Li J. Bawei chenxiang wan ameliorates cardiac hypertrophy by activating AMPK/PPAR-α signaling pathway improving energy metabolism. Front Pharmacol. (2021) 12:653901. 10.3389/fphar.2021.653901 34149410 PMC8209424

[73] Abudureyimu M Yu W Cao RY Zhang Y Liu H Zheng H. Berberine promotes cardiac function by upregulating PINK1/Parkin-mediated mitophagy in heart failure. Front Physiol. (2020) 11:565751. 10.3389/fphys.2020.565751 33101051 PMC7546405

[74] Liu BY Li L Liu GL Ding W Chang WG Xu T. Baicalein attenuates cardiac hypertrophy in mice via suppressing oxidative stress and activating autophagy in cardiomyocytes. Acta Pharmacol Sin. (2021) 42:701–14. 10.1038/s41401-020-0496-1 32796955 PMC8115069

[75] Pillai VB Samant S Sundaresan NR Raghuraman H Kim G Bonner MY. Honokiol blocks and reverses cardiac hypertrophy in mice by activating mitochondrial Sirt3. Nat Commun. (2015) 6:6656. 10.1038/ncomms7656 25871545 PMC4441304

[76] Gao J Zhang K Wang Y Guo R Liu H Jia C. A machine learning-driven study indicates emodin improves cardiac hypertrophy by modulation of mitochondrial SIRT3 signaling. Pharmacol Res. (2020) 155:104739. 10.1016/j.phrs.2020.104739 32135248

[77] Ramalingam A Budin SB Mohd Fauzi N Ritchie RH Zainalabidin S. Targeting mitochondrial reactive oxygen species-mediated oxidative stress attenuates nicotine-induced cardiac remodeling and dysfunction. Sci Rep. (2021) 11:13845. 10.1038/s41598-021-93234-4 34226619 PMC8257608

[78] Li W Yin L Sun X Wu J Dong Z Hu K. Alpha-lipoic acid protects against pressure overload-induced heart failure via ALDH2-dependent Nrf1-FUNDC1 signaling. Cell Death Dis. (2020) 11:599. 10.1038/s41419-020-02805-2 32732978 PMC7393127

[79] Meng G Liu J Liu S Song Q Liu L Xie L. Hydrogen sulfide pretreatment improves mitochondrial function in myocardial hypertrophy via a SIRT3-dependent manner. Br J Pharmacol. (2018) 175:1126–45. 10.1111/bph.13861 28503736 PMC5866985

[80] Huang L Chen R Liu L Zhou Y Chen Z. Lactoferrin ameliorates pathological cardiac hypertrophy related to mitochondrial quality control in aged mice. Food Funct. (2021) 12:7514–26. 10.1039/D0FO03346D 34223567

[81] Ma M Chen W Hua Y Jia H Song Y Wang Y. Aerobic exercise ameliorates cardiac hypertrophy by regulating mitochondrial quality control and endoplasmic reticulum stress through M(2) AChR. J Cell Physiol. (2021) 236:6581–96. 10.1002/jcp.30342 33615478

[82] An D Zeng Q Zhang P Ma Z Zhang H Liu Z. Alpha-ketoglutarate ameliorates pressure overload-induced chronic cardiac dysfunction in mice. Redox Biol. (2021) 46:102088. 10.1016/j.redox.2021.102088 34364218 PMC8353361

[83] Mohammed SA Paramesha B Meghwani H Kumar Reddy MP Arava SK Banerjee SK. Allyl methyl sulfide preserved pressure overload-induced heart failure via modulation of mitochondrial function. Biomed Pharmacother. (2021) 138:111316. 10.1016/j.biopha.2021.111316 33684689

[84] Lucas AMB de Lacerda Alexandre JV Araújo MTS David CEB Ponte Viana YI Coelho BN. Diazoxide modulates cardiac hypertrophy by targeting H2O2 generation and mitochondrial superoxide dismutase activity. Curr Mol Pharmacol. (2020) 13:76–83. 10.2174/1874467212666190723144006 31340743

